# A case presented with “as if” phenomenon

**DOI:** 10.4103/0019-5545.37673

**Published:** 2007

**Authors:** Soumitra Ghosh, Kushal K. Tamuli, Sabita Dihingia

**Affiliations:** Department of Psychiatry, Assam Medical College and Hospital, Dibrugarh, Assam, India

**Keywords:** “As if” phenomenon, depersonalization, derealization

## Abstract

A 22-year-old young male who was brought with a history of feeling of unreality, as if he himself and his surroundings had changed, since six months without any comorbid mental disorder or physical illness. Premorbidly he had some “obsessional traits”. Mental state examination revealed perceptual disorder in the form of depersonalization and derealization with insight grade VI. The patient was admitted and treated with pharmacotherapy and behavior therapy (relaxation exercise).

In “as if” phenomenon, the affected person is not delusionally convinced about the alteration of the perception of self and external world. It is usually found in depersonalization and derealization, which may be primary or secondary. Transient experiences of depersonalization and derealization are extremely common in normal and clinical populations. They are the third most commonly reported psychiatric symptoms after depression and anxiety and usually occur in association with other mental and substance-use disorders, although some authors conceptualize depersonalization disorder as a distinct disorder with a characteristic course that is independent of mood, anxiety and personality.[1] However, primary depersonalization is thought to be a rare phenomenon.[2]

## CASE REPORT

A 22-year-old young boy, with 14 years of formal education, working as a postman, from a nuclear family of lower middle socioeconomic status, who was leading a happy and peaceful life six months ago presented to our outpatient department in August 2005 with a history of feeling of unreality, as if he himself and his surroundings had changed, and heaviness of head.

Suddenly in the first week of March 2005 while he was playing with his friends, he felt that they were changed; they were not his friends - as if they were ghosts or devils. He was scared and ran to his home; he felt his house, even his mother also, was changed, These feelings continued and he often became fearful and restless, as he could not distinguish between the real and the unreal. He continued his job although he had decreased interest in sociocultural activities. While he went to the market or any other crowded place, he felt as if he was roaming in some different world or in a dream. He often pinched himself and felt the pain. He realized that these feelings were his own, wanted to get rid of these but could not. Gradually his sleep became disturbed; he would go to bed late at night and awaken late in the morning with complaint of feeling heaviness in his head. He had psychiatric consultation and was on medication for four months prior to his admission to our department. Improvement was negligible. The patient reported that he had the same experience once, when he took *bhang* for the first time one month prior to this illness, which lasted for a few hours and subsided spontaneously. There was no history of head injury, seizure, substance abuse or abnormal behavior. His childhood history was uneventful, with normal developmental milestones. There was no significant past or family history. Premorbidly the patient had few obsessional traits. Thorough physical examination was done, which revealed no abnormality. On mental status examination, the patient appeared anxious but otherwise conscious, cooperative, well groomed with adequate eye contact; and rapport could be easily established during the interview. His speech was relevant and coherent, and there was no evidence of formal thought disorder. However, he was preoccupied with his illness and distressed with the perceptual disturbance in the form of depersonalization and derealization. Judgment and insight was intact.

### Investigation

Random blood sugar (RBS), thyroid function test, Electroencephalogram (EEG), computed tomography (CT) scan were done, which were within normal limits.

The case was diagnosed as *Depersonalization Derealization Syndrome* as it fulfilled all the criteria given in ICD-10 and was treated with selective serotonin reuptake inhibitor (SSRI) (Paroxetine 25 mg/day), along with behavioral therapy (daily relaxation exercise).

## DISCUSSION

Depersonalization derealization syndrome involves an unpleasant chronic and disabling alteration in the experience of self and environment. In addition to these classic features of depersonalization and derealization, symptoms may encompass alteration in bodily sensation and a loss of emotional reactivity. There may be a sensation of being an outside observer of one's mental processes, one's body or part of one's body.[3] Depersonalization derealization syndrome is primarily a disturbance in the integration of perceptual experience and being distressed by it. Lifetime prevalence of depersonalization derealization syndrome in the community is unknown. A population-based survey using diagnostic interviews showed prevalence rate of clinically significant depersonalization derealization in the range of 1-2%.[4] Onset is usually during adolescence or early adulthood, with a male-female ratio of 1:2.

The relationship of depersonalization and derealization with certain other psychiatric disorders suggests possible common pathophysiological factors. Patients with depersonalization are often anxious, but it is an open question whether anxiety leads to depersonalization or vice versa.[5]

Depersonalization and derealization phenomenon also occurs in conjunction with substance abuse like alcohol, cannabis, barbiturates, beta blockers and benzodiazepines.[6] Depersonalization and derealization as a symptom are uncommon and transient in cannabis intoxication, particularly when used by naïve subjects, but resolve within few hours.[7] However, in our case the subject was not only a naïve user but the symptoms refused to disappear with time (six months), which makes it all the more likely to be a case of primary depersonalization derealization syndrome.

Primary depersonalization derealization syndrome is probably more common than previously thought.[8] Baker *et al* carried out a large and comprehensive clinical and psychopathological survey of a series of patients who made contact with a research clinic. Out of 204 cases, they found that as many as 71% of their study sample met the criteria for primary depersonalization disorder.[9]

There is as yet no definitive established treatment for depersonalization derealization syndrome. There is some systematic evidence that antidepressants (SSRIs) may be helpful.[10] Depersonalization was significantly more likely to improve if comorbid anxiety disorder improved.[11] Many different types of nonpharmacological therapies have been used to treat this condition.[12–14]

Lamotrigine, tried in an open trial in patients with depersonalization derealization, is quite encouraging in chronic cases. It was more effective when combined with antidepressant. Lamotrigine acts as an “add-on” treatment in depersonalization.[15] Mood stabilizers, typical and atypical antipsychotics or anticonvulsants can be used.[3]

## CONCLUSION

As primary depersonalization derealization syndrome is a rare presentation with poor prognosis, very few studies have been done on this subject. It is hoped that further studies will enable us to know more about this in future.
